# Dynamic Mechanical Analysis and Thermal Expansion of Lignin-Based Biopolymers

**DOI:** 10.3390/polym13172953

**Published:** 2021-08-31

**Authors:** Simona-Nicoleta Mazurchevici, Dorin Vaideanu, Doreen Rapp, Cristian-Dragos Varganici, Constantin Cărăușu, Mihai Boca, Dumitru Nedelcu

**Affiliations:** 1 Department of Machine, Manufacturing Technology, “Gheorghe Asachi” Technical University of Iasi, Blvd. Mangeron, No. 59A, 700050 Iasi, Romania; simona0nikoleta@gmail.com (S.-N.M.); c_carausu@yahoo.com (C.C.); mihaitzaboca@yahoo.com (M.B.); 2 Faculty of Physics, “Alexandru Ioan Cuza” University, 11 Carol I Blvd., 700506 Iasi, Romania; naturaone@gmail.com; 3 Applications Laboratory, NETZSCH-Gerätebau GmbH, Wittelsbacherstraße 42, 95100 Selb, Germany; doreen.rapp@netzsch.com; 4 Centre of Advanced Research in Bionanoconjugates and Biopolymers, “Petru Poni” Institute of Macromolecular Chemistry, 41A Aleea Grigore Ghica-Voda, 700487 Iasi, Romania; varganici.cristian@icmpp.ro; 5 Mechanical Engineering Department, Technical Sciences Academy of Romania, 030167 Bucharest, Romania

**Keywords:** dynamic mechanical analysis, thermal expansion, thermogravimetric analysis, lignin-based biopolymers

## Abstract

Biodegradable materials investigation has become a necessity and a direction for many researchers worldwide. The main goal is to find sustainable alternatives which gradually replace plastics based on fossil resources from the market, because they are very harmful to the environment and to overall quality of life. In order to get to the stage of obtaining different functional parts from biodegradable materials, it is necessary to study their properties. Taking into account these shortcomings, this paper aims at the mechanical characterization (DMA—Dynamic Mechanical Analysis) and thermal degradation (thermogravimetric analysis (TGA)) of lignin-based biopolymers: Arboform LV3 Nature^®^, Arboblend^®^ V2 Nature, and Arbofill^®^ Fichte Arboform^®^ LV3 Nature reinforced with aramid fibers. The tested samples were obtained by using the most common fabrication technique for polymers—injection molding. The obtained results for the DMA analysis showed separate polymeric-specific regions for each material and, based on the tanδ values between (0.37–0.54), a series of plastics could be proposed for replacement. The mechano-dynamic behavior could be correlated with the thermal expansion of biopolymers for temperatures higher than 50/55 °C, which are thermally stable up to temperatures of at least 250 °C.

## 1. Introduction

Due to the increasingly obvious climate change caused by the use and burning of fossil fuel products (made of rapidly depleting resources), a worldwide great interest from academia, industry, governments, to name a few sectors, has emerged, which aims to support the environment by using vegetal biomass as a raw material that presents the potential to reduce greenhouse gas emissions [1].

Lignin is an extremely abundant aromatic biopolymer material; the annual quantity produced by the pulp and paper industry as a by-product is about 50 million tons, which is often burned in order to generate heat and electricity or is used in low-value applications, [2,3]. However, in recent decades, the concern of both scientists and the general public about environmental pollution has begun to increase, so they have become more aware of the value provided by lignin, which considered a waste product until recently. Nowadays, lignin is one of the most sustainable and essential bio-resources widely used for the production of nanomaterials, carbon fibers, and improved composites, offering advantages that are both economical (stimulate local and regional economies through employment opportunities in biomass production and processing; products that can be obtained at a lower cost) and related to the safety and quality of the environment. Moreover, it is estimated that in the near future, the usable amount will double, since the substitution of petroleum products with renewable fuels and chemicals from lignocellulose biomass is a global level goal [2,3,4,5].

Lignin makes up 15% to 35% of the cell walls of terrestrial plants and it behaves like a permanent binding agent between cells, leading to energetic storage and the retention of a large amount of atmospheric carbon, acting as a UV blocker, an antioxidant, and a hydrophobic agent in plants. After plant death, lignin undergoes natural biodegradation with the help of soil microorganisms, resulting in the formation of soil organic matter. Thereby, lignin has an important impact on vascular plant physiology, the carbon cycle in nature, and the ecological balance of the Earth.

According to the literature, lignin-containing polymers present several specific characteristics, such as high thermal stability, biodegradability, antioxidant properties, antimicrobial actions, adhesive properties, etc., which allows their extensive use in a wide range of industrial applications and also their compatibility with specific applications [5,6,7,8].

In this manuscript, thermal and mechanical properties of injected samples from thermoplastic materials that are part of the polymers category based on renewable resources are studied, as they have an increased rate of biodegradability. These biopolymers were developed and marketed by Tecnaro GmbH in collaboration with the Fraunhofer Institute for Chemical Technology, being part of three different material families: Arboform^®^ “liquid wood”, Arboblend^®^ wood-plastic composites and Arbofill^®^ biopolymer compounds [9].

In order to obtain parts from biopolymers by means of injection molding, no intervention or improvement is required on the equipment, but more attention must be paid to establishing the process parameters, especially on the injection temperature and pressure. For better stability of the process, reinforcing materials can be introduced, which will facilitate the injection but will also increase the material properties.

The chosen biopolymers for this manuscript can be used in different fields, being able to yield landmarks/functional products (such as for the automotive industry) but also products that are more related to aesthetic items, such as furniture, toys, musical instruments, etc.

The novelty of the manuscript is that involves obtaining information about some mechanical and thermal characteristics of three lignin-based biopolymers, Arboform^®^ LV3 Nature^®^, Arboblend^®^ V2 Nature, and Arbofill^®^ Fichte; currently, there is not extensive research about these materials, and from to the obtained results, their ability to successfully replace a series of conventional plastic materials is proven. Additionally, the study considers an older problem of the present research group, related to some products obtained from the studied materials that showed a significant expansion after a certain longer use time. Following the undertaken/performed analyses, conclusions could be drawn on the behavior in the use of biopolymer products.

## 2. Materials and Methods

In the present study, the used materials were polymer-based on renewable raw materials which, according to the biopolymer company—Tecnaro GmbH (Ilsfeld, Germany), have the following structure profiles: Arboform^®^, known commercially as “liquid wood”, is a lignin-based polymer; it is only composed of renewable raw materials and thus is 100% biodegradable. Additionally, the materials incorporated in the lignin matrix include natural fibers (fiber plants) such as hemp and flax, and natural additives (wax, resins, etc.). The Arboblend^®^ polymer combines various property profiles and is up to 100% bio-based. Depending on customer requirements, its properties vary, as does its chemical formula; it is capable of being composed of a wide range of biopolymers: polylactic acid, polycaprolactone, polyhydroxyalkanoates, polyester, lignin, starch, cellulose, natural resins and waxes and vegetable fibers. Arboblend^®^ has similar mechanical properties to, for example, ABS (acrylonitrile butadiene styrene). Arbofill^®^ is a mixture of renewable raw materials (up to 80%) and plastics [8]. These three thermoplastic groups were developed in order to reduce the consumption of fossil raw materials (which are found in finite quantities) by replacing them with renewable raw materials available in the environment in industrial quantities. The studied materials can be processed by using conventional technologies such as injection molding or profile extrusion, but also modern technologies, such as 3D printing (the Fused Deposition Modeling method) and others.

The main properties of the studied materials are presented in Table 1.

For the DMA method, the analyses were realized by using PerkinElmer Diamond Dynamic Mechanical Analysis (UK), Pyris 7.0 software with the Dual Cantilever test method, according to D5418 DMA. The dimensions of the tested samples were: length—20 mm, width—10 mm, thickness—4 mm. The sample was run at 2 °C/min, in the −85–180 °C temperature range, depending on the material behavior, with a dynamic force of 5 N, deformation of 50 µm, and a frequency of 1 Hz *Tg*, which was determined from the peak of the *tanδ* curve, and the E′, E″ curves were represented in a logarithmic coordinate system in order to better highlight the variations recorded during the heating and bending of biopolymers.

The DMA samples were obtained by using injection molding technology, with the main technological parameters highlighted in Table 2.

The thermal expansion of the injected samples was determined in collaboration with NETZSCH Applications Laboratory (Thermophysical Properties Section) by using an NETZSCH model DIL 402 Expedis Select pushrod dilatometer (NETZSCH-Gerätebau GmbH, Selb, Germany). The dilatometer was equipped with a copper furnace capable of operation between 180–500 °C. The system was vacuum tight, allowing measurements to be carried out in pure inert or oxidizing atmospheres, as well as under vacuum. A set of primary standards, including fused silica, sapphire, platinum, tungsten, etc., is available for the length calibration. The expected expansion of the specimen and the temperature range of the measurement, of course, dictate which standard should be used. Data acquisition and evaluation, as well as instrument control, are accomplished with an MS Windows™ Thermal Analysis software package. The software includes semiautomatic routines for correction of the sample holder expansion, as well as computation of the expansion coefficients, onset and peak temperatures, inflection points, rate of expansion, etc. The dilatometer DIL 402 Expedis Supreme/Select is based on national and international standards, such as ASTM-D 696, -E 228, -E 831.

The dilatometer measurements were carried out using the following parameters: sample holder—SiO_2_; sample thermocouple—type K; pushrod force—0.05 N; atmosphere—He at 40 mL/min; temperature program—−150 °C to 120 °C at 10 K/min; reference sample—Al_2_O_3_; sample length—~25 mm.

Thermogravimetric analysis was performed using TGA NETZSCH STA 449 F1 Jupiter equipment (NETZSCH-Gerätebau GmbH, Selb, Germany) with a TG thermocouple, Proteus 6.0 software. The mass of the samples was in the range of 40–45 mg and was heated with a rate of 10 K/min, carrier gas—nitrogen (99.999% purity) with 250 mL/min flow rate, 25–700 °C temperature range. The samples were heated in an open Al_2_O_3_ crucible and an empty crucible was used as the reference material.

The structural and morphological analysis of the Arboform^®^ LV3 Nature reinforced with aramid fibers material was performed in order to highlight the structural uniformity that resulted from the reinforcement of the Arboform^®^ LV3 Nature-based polymer with aramid fibers in a percentage of 15%. The analyses performed in this sense were: FT-IR for chemical composition, and SEM and EDX for structure and morphology.

Scanning Electron Microscopy (SEM) and Energy-dispersive X-ray spectroscopy (EDX) were carried out using a QUANTA 200 3D SEM-FIB electron microscope (FEI Company, Fremont, CA, USA). The images were obtained taking into account the following parameters: the acceleration voltage of the secondary electrons—30 Kv; magnification power 100X–5000X; working distance—15 mm; LFD detector (Large Field Detector); tilt angle of 0°; the pressure inside the microscope chamber—60 Pa.

The FT-IR (Fourier transform infrared spectrometer) spectrum was registered on a Bruker-Vertex 70 (Brüker, Germany) apparatus equipped with a MIRacleTM ATR accessory provided with diamond crystal plate with a 1.8 mm diameter in the range of (4000–600) cm^−1^ with 4 cm^−1^ resolution. The *FT-IR* (Fourier transform infrared spectrometer) spectrum was recorded using a photoluminescence spectrometer, FLS980 Edinburgh (Edinburgh Instruments, Livingston, Scotland), in the range of 4000–600 cm^−1^ with 4 cm^−1^ resolution and scan rate of 32. The spectrophotometer was equipped with a MIRacle^TM^ ATR accessory designed for single or multi-reflection attenuated total reflectance (ATR), a diamond plate.

## 3. Results and Discussion

### 3.1. DMA Analyses

The results of the DMA analyses for Arboform^®^ LV3 Nature, Arboblend^®^ V2 Nature, Arbofill^®^ Fichte, and Arboform^®^ LV3 Nature reinforced with aramid fibers are presented below. In order to improve the functional characteristics of the Arboform^®^ LV3 Nature material, which contains the highest amount of renewable raw material (100%) compared to those selected for the present study, the reinforcement with 15% aramid microfibers, of the Kevlar type, was additionally made without modifying the biodegradability. Additionally, the addition of reinforcement increased the material processability.

**Figure 1:**
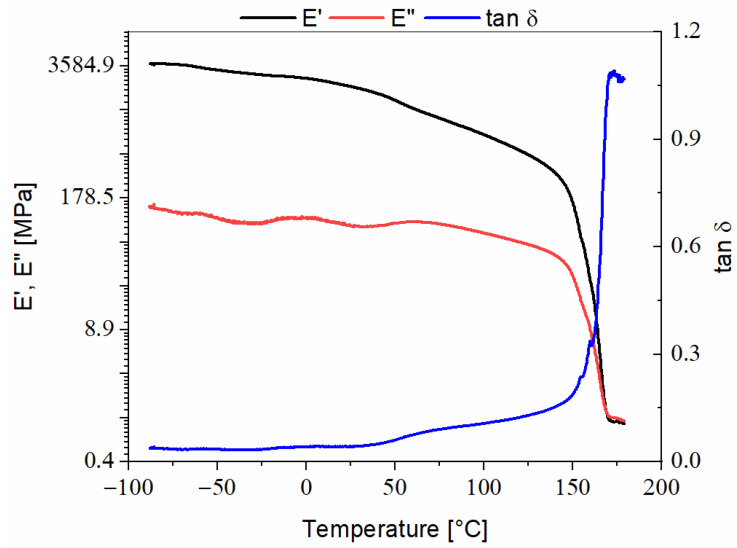
DMA thermogram recorded during heating of Arboform^®^ LV3 Nature.

Arboform^®^ LV3 Nature, Figure 1—The DMA thermogram of the Arboform^®^ LV3 Nature biodegradable material highlighted the following aspects:

- On the damping curve, there is an increase around the temperature of 140 °C, which can be attributed to the beginning of material melting [11], and the intersection point between the curve E′ (storage modulus) and tanδ (damping/loss factor) is associated with the glass transition of the Arboform^®^ LV3 Nature material, at a temperature of 54.5 °C, which is also confirmed by previous works, [12]. Following the thermogram, it can be observed that there is no sudden increase in the damping curve; its value increases with the test temperature. However, it should be noted that according to a previous study, [12], which performed the DMA analysis using the three-point bending method, the average value of the tanδ was about 0.33 at 67 °C, [12], a value that can be assimilated, but also, one must consider the different method of analysis used in the present study;
- Regarding the storage modulus and loss modulus (E″) curve shapes, inclined descending slopes denote the fact that the transition to the viscous state was made slowly. This behavior is also supported by the shape of the internal friction curve. The values of stored energy and dissipated energy are approximately equal, with slightly higher values for E′, denoting the material heterogeneity.

**Figure 2:**
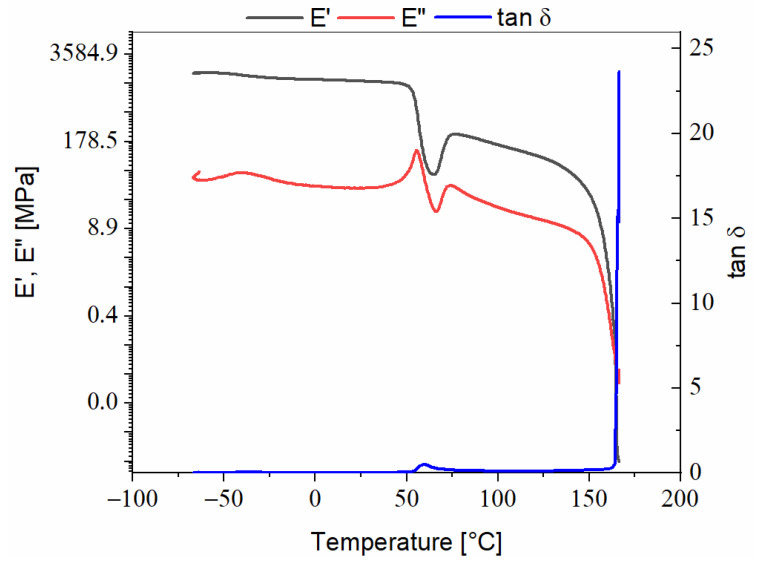
DMA thermogram recorded during heating of Arboblend^®^ V2 Nature.

Arboblend^®^ V2 Nature, Figure 2:

- The storage modulus values are very high at beginning of the experiment, approximately 1900 MPa, because the macromolecules are found in a frozen state, so the mobility of the segments is constrained;
- In the field of positive values, with the decrease in the storage modulus values, the beginning of α-type relaxation may be observed, marked around the 60 °C temperature;
- The maximum damping value, 0.50 (at 60 °C), in the α-type relaxation region is also the region in which the glass transition of the Arboblend^®^ V2 Nature material takes place;
- Around the 155 °C temperature on the tanδ curve, there is an increase which is attributed to the beginning of material softening (liquid flow), which also, according to previous calorimetric determinations of the research group, [13], has a melting point at 158 °C. Additionally, with the beginning of material softening, the abrupt decrease in E′ and E″ curves takes place, as a plasticizing effect of melting and of high temperature—the macromolecular chains of Arboblend^®^ V2 Nature start to slide side by side and generate the flowing phenomenon in the flowing (terminal) region. Thus, the mechanical analysis in dynamic regime cannot be performed further;
- The storage modulus curve decreases suddenly, almost vertically, which indicates that the phase change takes place in a very restricted time and temperature interval, denoting the material homogeneity which during the test behaves unitarily, an aspect confirmed by the maximum peak of the tanδ curve, which has a sharp and narrow shape;
- The plateau region, which precedes the α relaxation region, characterized by structural reorganization phenomena, takes place in the (78–155) °C temperature range;
- The loss modulus curve is highlighted on the graph at much lower values than those of the storage modulus, outlining the elastic behavior of the material, because during testing, it stored more energy than it lost.

**Figure 3:**
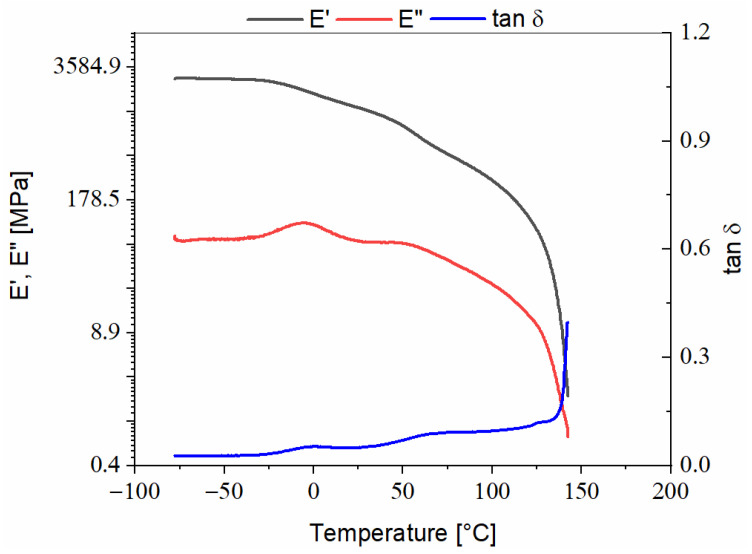
DMA thermogram recorded during heating of Arbofill^®^ Fichte.

Arbofill^®^ Fichte, Figure 3:

- For the Arbofill^®^ Fichte material, the thermogram highlights the appearance of a sharp peak on the damping curve at a temperature of 144.6 °C that can be associated with its melting point, also reported by other authors [14]. The glass transition of the material is considered, in the case of this material, to be at the intersection point of the storage modulus and damping curves; therefore, at a temperature of 58.58 °C, the tanδ is approximately 0.07;
- Regarding the shape of the storage modulus and loss modulus curves, this denotes the fact that the phase transitions appear slowly but also the fact that the values E′ and E″ are approximately equal, so the material has an elastoplastic behavior.

**Figure 4:**
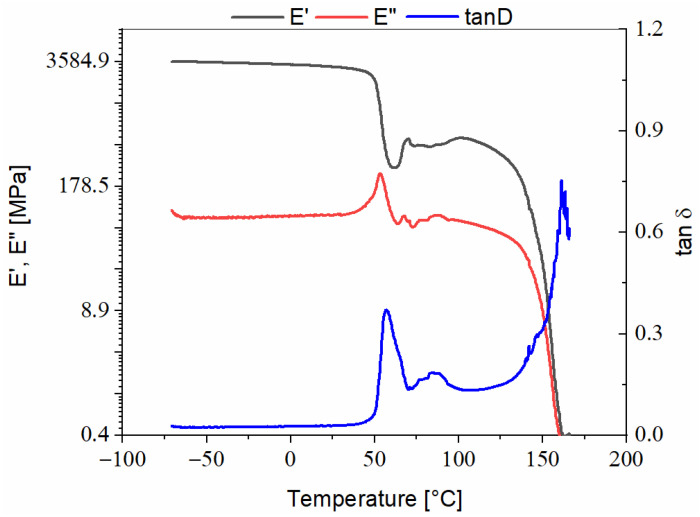
DMA thermogram recorded during heating of Arboform^®^ LV3 Nature reinforced with aramid fibers.

Arboform^®^ LV3 Nature reinforced with aramid fibers, Figure 4:

- On the damping curve, a representative peak with a maximum value of 0.37 appears at a temperature of 57 °C in the α relaxation region, the temperature at which Tg (glass transition) of the reinforced material takes place. A second, much lower peak can be observed with a centered tanδ at 0.18 and a temperature of 86.52 °C, which can be associated with a transformation that occurs in the material structure during its progressive heating, most likely to be water evaporation and/or the decomposition of natural additives or dyes [15]. On the damping curve, no transition that could be associated with the reinforcement—aramid fibers—is noticed, as it is thermally stable up to 450 °C [16], well above the range set for this analysis, and the glass transition of the aramid fibers appears in the (345–360) °C temperature range, [17]; otherwise, there are only losses of hydrogen bonds;
- On the damping curve, around the 145 °C temperature mark, an ascending slope appears, which indicates the beginning of the material softening—the flowing zone— which according to reference [18], has a melting point at 171 °C;
- The damping peak is narrow and sharp and the descending E′ slope is steep, which indicates that the chemical structure of the material is homogeneous, and the transition takes place in a short time and temperature range;
- The rubbery plateau region is in the 96–148 °C temperature range.

Comparing the four analyzed biodegradable materials, from the highlighted mechanical response point of view, it can be concluded that Arboform^®^ LV3 Nature and Arbofill^®^ Fichte are semi-crystalline materials, as they have an alpha star (Tα*) transition that appears on the “rubbery plateau”. These transitions, according to the literature, [19], are associated with sliding between crystallites and help to extend the polymer operating range above the temperature at which the vitreous transition occurs. This transition, Tα*, is extremely sensitive and is significantly influenced by the variations induced during processing, and can be oscillated (decreased or increased) by changes in processing conditions, the temperatures used and also by physical aging. Additionally, the α* transition is used by manufacturers to optimize the material properties. The semi-crystalline structures of the materials are attested by XRD (X-ray Diffraction) analyses performed and published previously, [13,17,20].

The Arboblend^®^ V2 Nature and Arboform^®^ LV3 Nature polymers reinforced with aramid fibers are semi-amorphous/amorphous polymers [12,13,21]. Both the Arboform^®^ LV3 Nature and Arbofill^®^ Fichte polymers show a viscoelastic behavior given by the storage and loss modulus curves, which are very similar in shape but also in values. The thermograms present a tendency of the storage modulus to decrease over the entire temperature range, caused by the progressive sliding of the macromolecular chains, eventually reaching the flow region.

For Arboblend^®^ V2 Nature and Arboform^®^ LV3 Nature reinforced with aramid fibers, the storage modulus curve (E′) highlights two significant changes that occur with increasing temperature: a sudden decrease in E′ in the range of 40–60 °C and a reduction in the rate of decrease in E′, starting with temperatures higher than 60 °C. The significant sudden decrease in the storage modulus is correlated with the relaxation of the amorphous phase, when the glassy state of the amorphous phase in the polymer matrix passes through its glass transition, followed by the accentuated decrease in E′. Additionally, an α-type relaxation appears on the curve of the loss modulus (E″), that can be attributed to the larger molecular chain segments in the amorphous phase that begin to move.

Comparing the tanδ values obtained for Arboblend^®^ V2 Nature and Arboform^®^ LV3 Nature reinforced with aramid fibers with that of other non-biodegradable polymers, it can be stated that, from this point of view, the materials that can be successfully substituted are: PP (polypropylene) and reinforcements of said PP with glass fiber, short glass fiber, nanoclay (tanδ—0.065, at 10 °C) [22]; 0.27 LDPE low-density polyethylene (at 50 °C), 0.24 LLDPE linear low-density polyethylene (at 40 °C), 0.28 HDPE (high-density polyethylene), (at 75 °C) [23]; blends of PP, tanδ—0.3–0.4—at around 130 °C) [24]; etc.

Additionally, making a comparison between the obtained results with those of other lignin-based biopolymers, it was observed that their viscoelastic behavior is relatively similar, as follows: for the PLA (polylactic acid) material, a maximum tanδ of about 0.7 was identified [25]; for lignin-based biodegradable polymer composites, different mixtures of PBS (Polybutylene succinate), lignin and PMDI (Polymeric diphenylmethane diisocyanate) with tanδ between 0.04–0.1 was identified [26]; for POM (Polyoxymethylene) blended with PLA in different percentage mass, tanδ in the 0.2 range was found [27]; polyamide 6.6 reinforced with short glass fiber (16%, 12%, 8%, 4%) had tanδ in the interval of 0.06–0.11 [28], and many more.

### 3.2. Thermal Expansion and Coefficient of Thermal Expansion of Biopolymeric Samples

**Figure 5:**
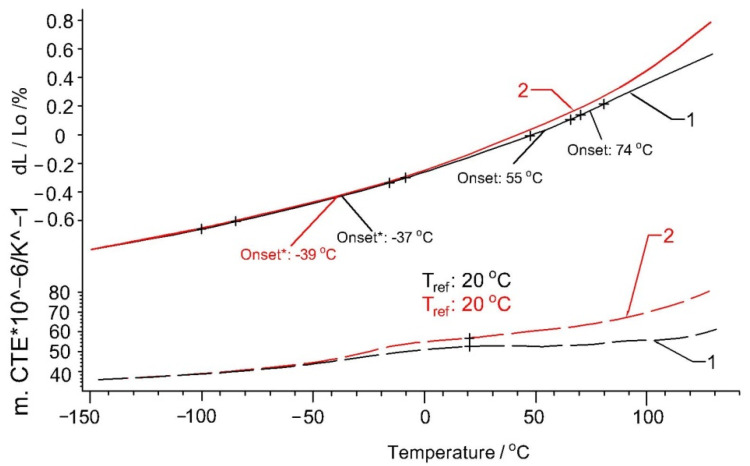
Thermal expansion (solid lines) and coefficient of thermal expansion of the sample Arbofill^®^ Fichte: 1—first heating; 2—second heating.

The results of the expansion measurements of the Arbofill^®^ Fichte material are shown in Figure 5. It is observed that the ∆L/L0 variation is approximately linear for longer temperature ranges. Non-linear variations may occur for small temperature ranges. There are two temperature ranges where some deviations are observed. This aspect can be explained by the structural changes inside the material which, by means of shortening the chemical bonds between the atoms, oppose the mechanical effects induced by expansion.

The thermal expansion (solid line) and the coefficient of thermal expansion (CTE, Tref.: 20 °C, dashed line) are depicted in the top and bottom part of the graph, respectively. In the beginning, the sample expanded continuously. Three steps were observed in the thermal expansion at −37 °C, 55 °C and 74 °C (extrapolated onsets). After this, the same sample was heated a second time. The thermal expansion of the second run was almost identical. It can be seen that the last two anomalies no longer existed for subsequent heating. The differences to the first run might be due to relaxation effects. At a temperature of −37/−39°C during thermal expansion, a β type relaxation effect is observed and was given by the local movement of C–H groups. For the 55–74 °C temperature range, an α -type relaxation effect is observed, which can be associated with transits in the vitreous phase. Moreover, this relaxation can be associated with a second-order phase transition, and most likely crystallization. In the 65–88 °C temperature range, Arbofill^®^ Fichte has an internal crystalline structure [14,29].

**Figure 6:**
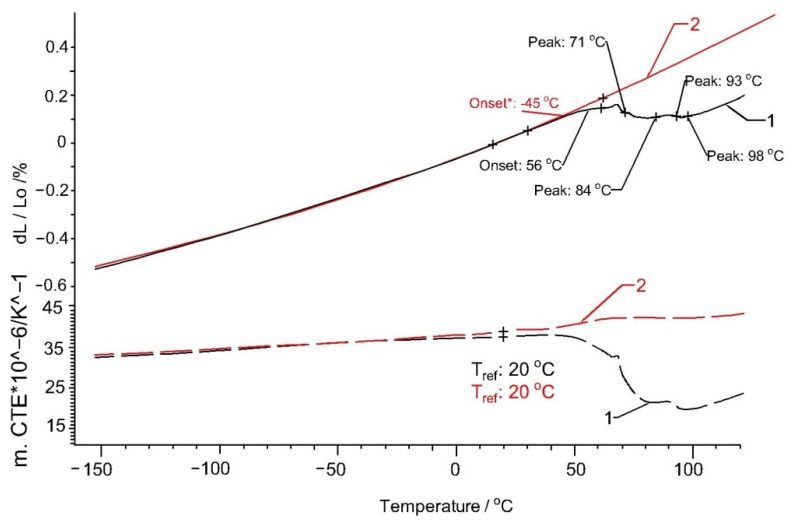
Thermal expansion (solid lines) and coefficient of thermal expansion of the sample Arboform^®^ LV3 Nature: 1—first heating; 2—second heating.

**Figure 7:**
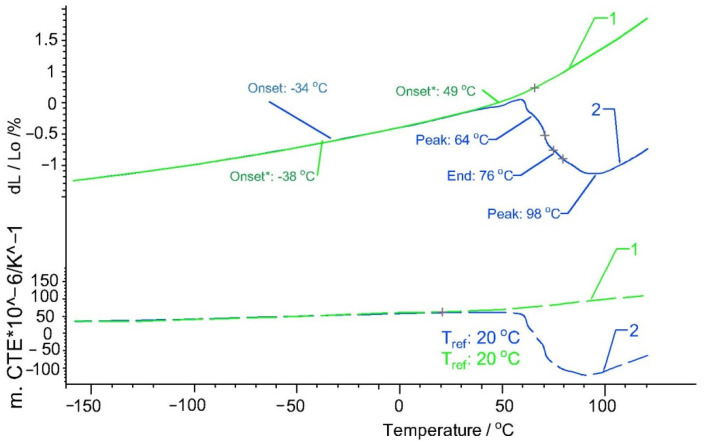
Thermal expansion (solid lines) and coefficient of thermal expansion of the samples Arboblend^®^ V2 Nature: 1—first heating; 2—second heating.

Figure 6 and Figure 7 show that, for both Arboform^®^ LV3 Nature and Arboblend^®^ V2 Nature, at the first heating, there is a contraction in the 45–100 °C temperature range with the decrease in the expansion coefficient. The cause of this phenomenon can also be a rearrangement (shrinkage) of the lignin matrix chemical bonds, which can also lead to a phase transformation in which the materials pass into the vitreous phase. These transformations are also highlighted in the graphs corresponding to the DMA-type mechanical tests.

**Figure 8:**
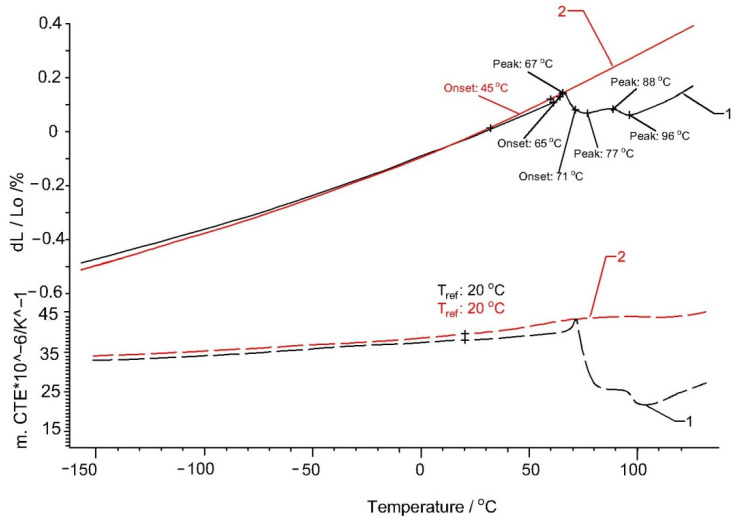
Thermal expansion (solid lines) and coefficient of thermal expansion of the samples Arboform^®^ LV3 Nature reinforced with aramid fiber: 1—first heating; 2—second heating.

For reinforced Arboform^®^ LV3 Nature, Figure 8 presents some changes in the temperatures at which the contractions take place. If for the pure biopolymer, the contractions start at a temperature of 45 °C, for the reinforced one, a first minor contraction takes place around the temperature of 37 °C, up to the temperature of 60 °C. After this temperature range, the material undergoes a linear expansion up to the temperature of 67 °C, after which it suffers the second higher contraction in two temperature ranges: 67–77 °C and 88–96 °C. After 96 °C, the material undergoes a linear expansion. For the second heating, it can be stated that the material behaves almost identically to unreinforced Arboform^®^ LV3 Nature.

A possible explanation would be that during the first heating, the fibers tend to oppose the contractions by modifying the temperature ranges, acting as a compensating agent. After the first heating, a uniform micro-dispersion of the fibers takes place next to the lignin matrix so that the material behaves approximately identically to the unreinforced Arboform^®^ LV3 Nature.

It can be seen that, for the second heating, the linearity of the expansion process is approximately respected. It is observed that Arbofill^®^ Fichte suffers the largest variation of dimensions for the same temperature range, and the expansions of all materials are in accordance with the variation of the thermal expansion coefficient.

The performed analyses also helped to establish the possible causes of the expansion of the injected parts from the studied materials after a certain period of operation. Thus, by analyzing the thermal expansion of the samples, it was observed that at the time of heating, the sample tends to lose its structural stability, by expansion, at temperatures above 55 °C. This change in the conformation of the biopolymer structure is also confirmed by the presence of the α relaxation process, identified during DMA analysis. The relaxation region α is most often in the case of polymers with an amorphous structure, [30,31], attributed to micro-Brownian motions that arise at the time of intervention of factors such as temperature rise or stress.

### 3.3. Thermogravimetric Analysis—TGA

**Figure 9:**
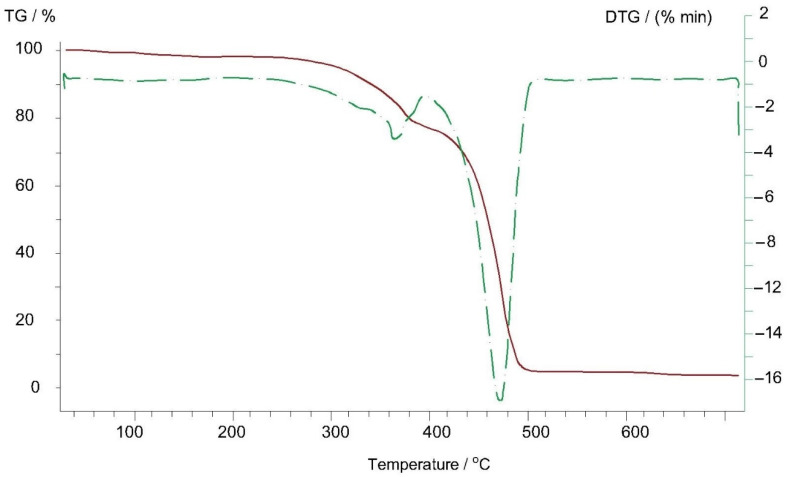
Thermogravimetric curve of the Arbofill^®^ Fichte sample.

**Figure 10:**
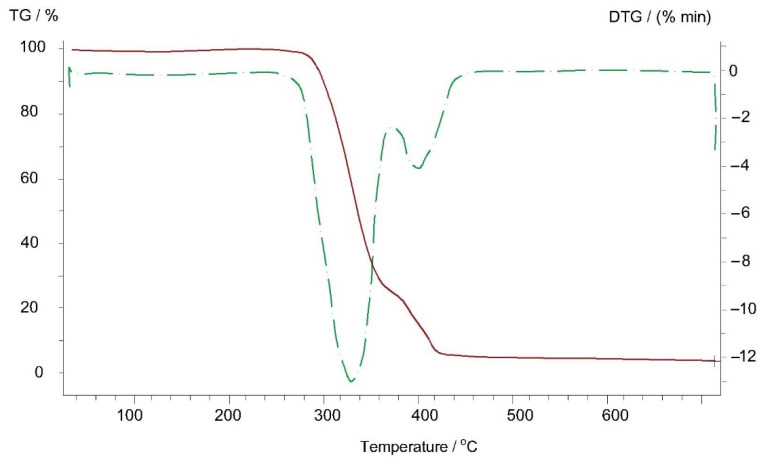
Thermogravimetric curve of the Arboblend^®^ V2 Nature sample.

**Figure 11:**
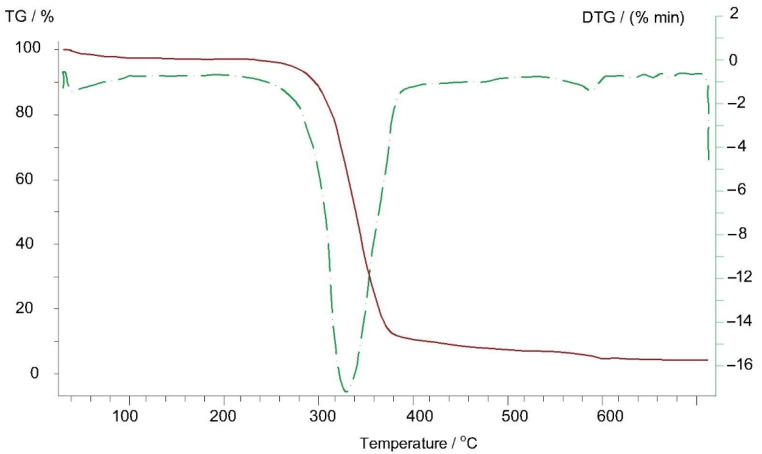
Thermogravimetric curve of the Arboform^®^ LV3 Nature sample.

**Figure 12:**
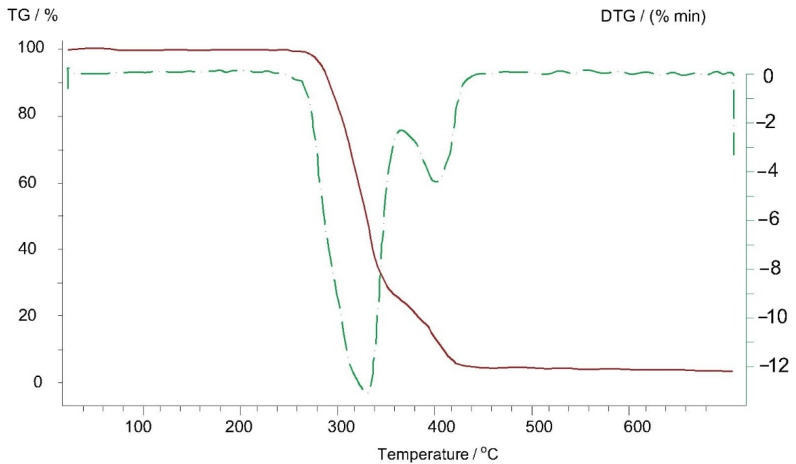
Thermogravimetric curve of the Arboform^®^ LV3 Nature reinforced with aramid fibers sample.

Thermogravimetric analyses of Arbofill^®^ Fichte, Arboblend^®^ V2 Nature, Arboform^®^ LV3 Nature and Arboform^®^ LV3 Nature reinforced with aramid fibers materials are presented in the following figures (Figure 9, Figure 10, Figure 11 and Figure 12). The main thermogravimetric characteristics obtained are presented in Table 3.

*Arbofill^®^ Fichte*, Figure 9: In the 89–139 °C temperature range, there is a first mass loss at a small percentage of only 1.33%, which can be associated with the beginning of water evaporation from the basic matrix of the material (lignin, natural fibers):

- The second mass loss at a percentage of 20.23%, in the 267–377 °C temperature range, coincides with the endothermic transformation that reaches the maximum phase at the temperature of 368 °C (T_max_ from DTG) and which is associated with the decomposition of a material constituent element, possible natural vegetable fibers or an additive/resin (binder);
- Significant mass loss, 71.76%, occurs starting at the temperature of 417 °C when there is complete destruction of the structure of the material base matrix due to its carbonization at a temperature of 463 °C, with the massive evaporation of water from the base matrix of the material;
- At the end of the thermal analysis, at a temperature of 699 °C, a residual mass of only 5.87% appears. The residue can be considered to be un-carbonized lignin because, according to the literature, it can withstand temperatures above 800 °C [32].

Arboblend^®^ V2 Nature, Figure 10: The thermogram of the Arboblend^®^ V2 Nature material made for the 20–700 °C temperature range includes two endothermic transformations that implicitly lead to weight loss:

- The first endothermic peak at a temperature of 332 °C is associated with the carbonization of the basic constituent of the material because, as it can be seen, in order to take place, this transformation requires the consumption of a larger amount of heat than in the case of the second endothermic peak. During this transformation, according to thermogravimetric measurements, a mass loss of 77% in the 278–354 °C temperature range was recorded;
- The endothermic minimum at 401 °C is associated with the degradation of a secondary material constituent, most likely a natural additive in the form of a resin. This transformation takes place with mass loss of a percentage of 21.08%, which is registered in the 379–419 °C thermal range;
- At a temperature of approximately 700 °C, a residual mass of 3.64% is found after the thermogravimetric analysis, much lower than in the case of the Arbofill^®^ Fichte material, also associated with the presence of lignin, which does not yet lose all microstructural bonds at this temperature.

Arboform^®^ LV3 Nature, Figure 11*:* At a temperature of 39 °C, an exothermic peak appears, which could be associated with the evaporation of a small percentage of water (according to the data provided by the manufacturing company, the humidity/water content is between 2–8%, [3]) from lignin that represents the matrix of Arboform^®^ LV3 Nature, or more likely the vaporization process of the natural additives, found as a percentage of 10% (shellac, flame retardant additives, resins). The transformation takes place with mass loss of only 2.15%:

- The minimum endothermic peak at the temperature of 335 °C is caused by the occurrence of the pyrolysis phenomenon (disintegration, deterioration) of the material base matrix, lignin, of up to 90%. Additionally, as expected during this transformation, there is a significant mass loss of 82.77%;
- At a temperature of 577 °C, there is a transformation that takes place with heat absorption and most likely is due to the decomposition and carbonization of a constituent element of the material, most probably an additive;
- At the same time, it can be seen that the residual mass (carbon) resulting from the pyrolysis of the lignin matrix is closely related to the percentage of lignin; the type of lignin molecule may have a greater or lesser number of carbon atoms;
- On the thermogravimetric curve of the Arboform^®^ LV3 Nature reinforced with aramid fibers, Figure 12, the following can be observed:
- At a temperature of 334 °C, an endothermic maximum is visible, which is attributed to the almost complete pyrolysis of the base material, with a mass loss in percentage of 83.67%;
- Another important weight loss of 2.24% is visible in the temperature range of 546–584 °C and is caused by the decomposition of a constituent of the biopolymer but also by the significant degradation of aramid fibers [33,34] used as material for reinforcing the Arbform^®^ V3 Nature biopolymer;
- At the end of the thermogravimetric analysis, at 700 °C, a residual mass of 10.55% remains, which can be largely associated with the lignin bonds that resisted up to this temperature but also with the aramid fibers which, according to the literature, degrade completely between the temperature of 700 and 900 °C, [34,35].

Comparing the thermograms recorded for the Arboform^®^ LV3 Nature base material with the one reinforced with aramid fibers, the following aspects are observed:

- The temperature associated with the evaporation of water and/or other constituents of the biopolymeric material increases by approximately 20 °C. Additionally, the maximum temperature at which most of the polymeric bonds are lost by thermal degradation, over 83%, increases by up to 9 °C when reinforcing Arboform^®^ LV3 Nature with aramid fibers;
- There is no improvement on the residual mass found at the end of the analysis because the thermal interval in which the aramid fibers lose a high mass weight, over 97%, is 480–520 °C [33,36]. Thus, at higher temperatures, it is impossible for this reinforcement to further improve the thermal characteristics of a polymer;
- The incorporation of aramid fibers, in a mass percentage of 15, increases not only the thermal resistance of the Arboform^®^ LV3 Nature biopolymer but also its mechanical characteristics, confirmed by the obtained results of the research groups in [12,37,38,39,40,41,42].

It is observed that the temperature at which the pyrolysis takes place is approximately the same for Arboform^®^ LV3 Nature, Arboform^®^ LV3 Nature reinforced with aramid fibers sample and Arboblend^®^ V2 Nature, but for Arbofill^®^ Fichte, it is approximately 120 °C higher. The occurrence of this phenomenon can be explained by the type of lignin used and the reinforcing materials that can lead to an increase in the pyrolysis temperature in the case of Arbofill^®^ Fichte. This aspect can be an indicator for the fields of applicability of each material.

### 3.4. Fourier Transform Infrared Spectroscopy—FT-IR

**Figure 13:**
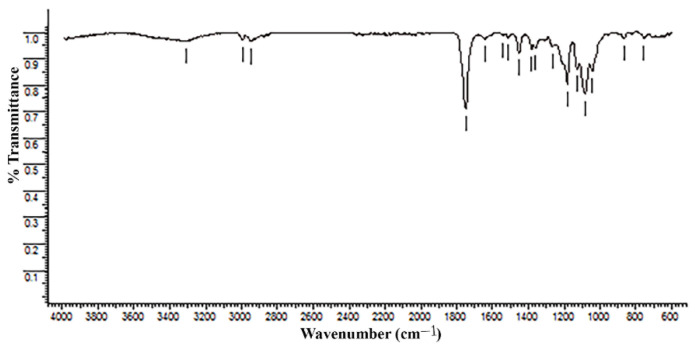
FT-IR spectra of Arboform^®^ LV3 Nature reinforced with aramid fibers.

To investigate the chemical structure of Arboform^®^ LV3 Nature reinforced with aramid fiber, FT-IR spectra, highlighted on Figure 13, were recorded. The composite biopolymer spectrum pointed out characteristic absorption bands which can be associated as follows:

- The most pronounced absorption band is recorded at 1750 cm^−1^ and is ascribed to the stretching vibrations of the ester carbonyl group (C=O from COOR groups). The bending vibration of this group appears at 1266 cm^−1^ and the deformation vibrations (C–OH) specific to secondary alcohols are visible at 1128 cm^−1^ [43,44,45]. All these three maxims are attributed to the presence of lignin and cellulose in the structure of the composite biopolymer;
- Another strong band is recorded at 1084 cm^−1^, corresponding to deformation of C–O–C anti-symmetrical stretching of polysaccharide [46] cellulose belonging to this category. Additionally, at 1181 cm^−1^_,_ a strong absorption band belonging to the C-O-C ester group is recorded [44];
- Additionally, the presence of lignin and cellulose, respectively, is highlighted by the peaks from 1452 cm^−1^ and 1382 cm^−1^, which are assigned to the asymmetric and symmetric bending deformation vibrations of δasCH3 and δsCH3, [43,44,47]. At 1361 cm^−1^, a deformation vibration of δOH is visible and [44] is attributed to the connections made between the biopolymer matrix and the embedded aramid fibers;
- The absorption band at 1313^−1^ indicates the incorporation of the cellulose in to the lignin matrix [48], and from 1043 cm^−1^, according to the scientific literature, coincides with the presence of hemicellulose and pectin [11];
- The low-intensity peaks from 1640 cm^−1^ (stretching vibration of –C=O, amide I band), 1512 cm^−1^ and 1542 cm^−1^ (curved vibration of –N–H) are closely related with the incorporation of aramid fibers into the lignin matrix [49,50,51,52,53];
- In the region between 3000 cm^−1^ and 2850 cm^−1^, wavelengths there are a series of four absorption bands (2852 cm^−1^, 2875 cm^−1^, 2945 cm^−1^ and 2996 cm^−1^) that are associated with aliphatic C–H stretching [43,44,45,54] due to the incorporation of natural additives in the Arboform^®^ LV3 Nature biopolymer. Additionally, in the literature, another natural additive present in the structure, canola oil, is visible at 824 cm^−1^, and may be relevant to this study [55];
- The absorption band from 3329 cm^−1^ is attributed to the hydrogen-bonded O-H stretching vibration of cellulose, present in all natural vegetable fibers [56];
- The bands at 867 cm^−1^ and 757 cm^−1^ are assigned to the amorphous and, respectively, crystalline phases of polylactic acid [45]. A weak signal is registered at 673 cm^−1^ and is attributed to the Si–O stretching vibrations, [57], which are most likely visible in the FT-IR analysis due to an impurity that appears during the sample manipulation.

### 3.5. Scanning Electron Microscopy—SEM and Energy Dispersive X-ray Analysis—EDX

SEM images were taken from the fracture surfaces of the samples in the case of both analyzed materials: Arboform^®^ LV3 Nature and Arboform^®^ LV3 Nature reinforced with aramid fibers.

**Figure 14:**
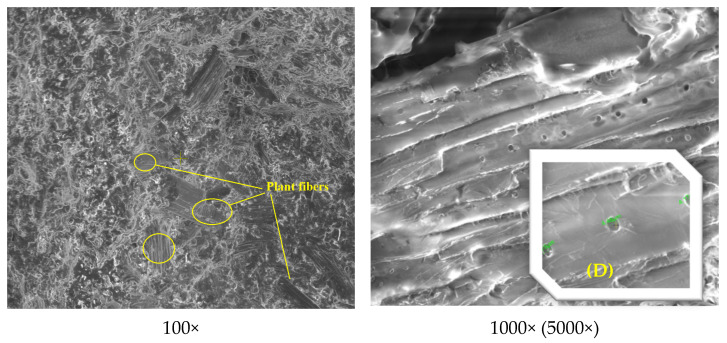
SEM images at different magnification powers for Arboform^®^ LV3 Nature.

Figure 14 shows the SEM images of the Arboform^®^ LV3 Nature biopolymer. According to the captured images, the analyzed surface has a uniform structure with unidirectional dendritic branches and small secondary dendrites. The natural plant (vegetable) fibers incorporated in the lignin matrix of the biopolymer can also be observed in a detailed image, with a porous structure and pores of micrometric dimensions, noted with (D). It must be specified that no de-bonding between lignin as the matrix and the natural fibers as reinforcement components was found in the breaking area of the sample.

**Figure 15:**
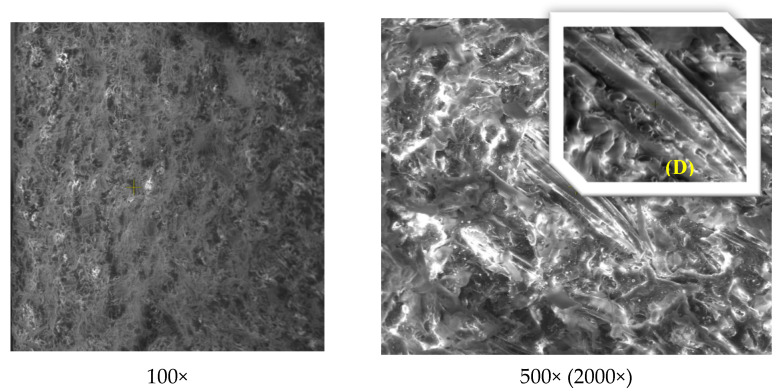
SEM images at different magnification powers for Arboform^®^ LV3 Nature Nature reinforced with aramid fibers.

Figure 15 highlights the presence of aramid fiber imprints in the biopolymer matrix, with good dispersion and interfacial bonding.

**Figure 16:**
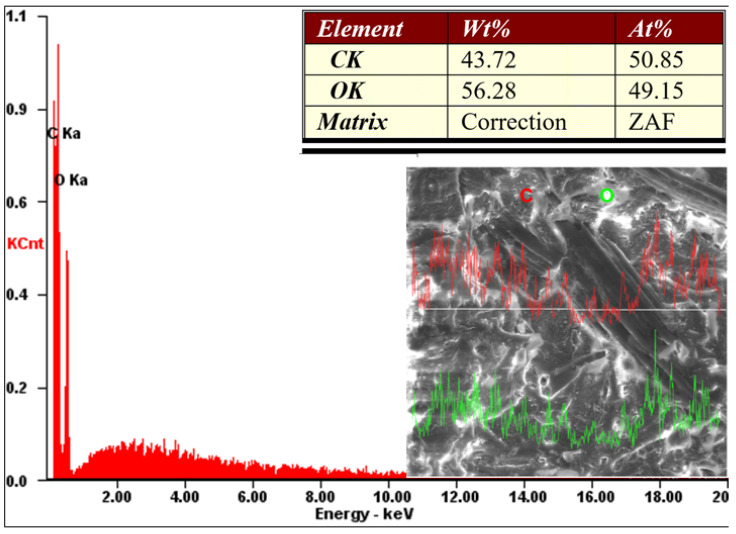
In-line EDX analysis for the injected sample from Arboform^®^ LV3 Nature, (kV: 30.00 Mag: 500×).

By X–Ray diffraction analysis of the pure Arboform^®^ LV3 Nature material, its constituent chemical elements were identified, as shown in Figure 16. The elements highlighted by the sample were oxygen and carbon in mass percentages of approximately 44% (carbon) and 56% (oxygen), with these elements being present in abundance in the structure of natural vegetable fibers, cellulose, hemicellulose, etc.

**Figure 17:**
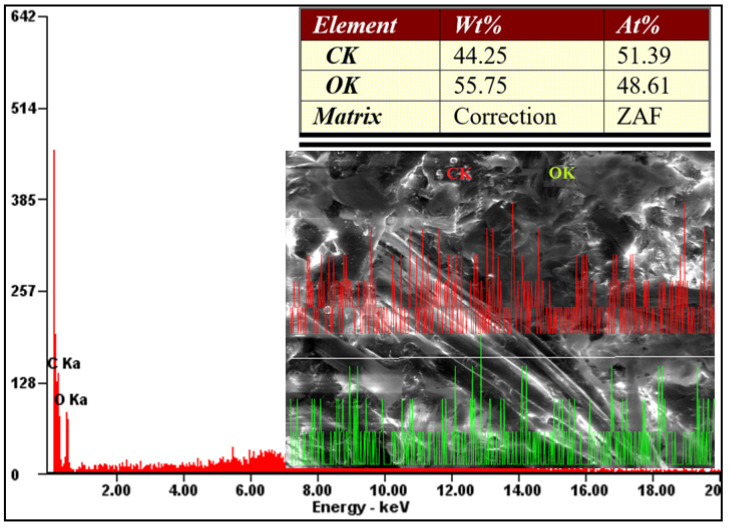
In-line EDX analysis for the injected sample from Arboform^®^ LV3 Nature ran forced with aramid fibers (kV: 30.00 Mag: 500×).

The sample from Arboform^®^ LV3 Nature, reinforced with aramid fibers (synthetic), has a chemical structure approximately similar to that of the pure biopolymer, as shown in Figure 17. This was influenced by the small amount (15%) of reinforcement introduced. Normally, according to the literature, aramid fibers of the Kevlar type—DuPont—are made by a condensation reaction of an amine and acid chloride, resulting an aromatic polyamide (para-aramid). The chemical elements involved in the aramid fibers’ structure are: hydrogen, azote, oxygen and carbon, [58]. However, in the EDX analysis performed, only the carbon and oxygen content were visible, most likely due to the too small amount of the other elements.

The aramid fibers used for the reinforcement of Arboform^®^ LV3 Nature are uniformly distributed in the biopolymer mass, without showing agglomerations, which is visible in Figure 15 and also Figure 17 (SEM capture in line). The reinforcement was used to improve the injection molding of the biopolymer but also to increase the characteristics, which was possible because the aramid consists of a long chain synthetic polyamide, which provides a very strong molecular structure, rigidity, resistance at wear, fire, organic solvent, etc. [59,60]. 

## 4. Conclusions

In addition to cellulose, lignin is the second major component of hard and softwood and annual plants such as herbs. As a by-product of the pulp and paper industry, lignin is available on an industrial scale and is gaining interest due to increasing environmental concerns and the depletion of petrochemical resources.

Following the performed analyses on the samples from biodegradable materials for mechanical analysis in a dynamic regime, it was found that the materials showed different behaviors depending on their structure, either viscoelastic or plastic behavior, with the values of the characteristics being comparable to those of common plastics. Thermal expansion and the coefficient of thermal expansion strengthened the results obtained from the DMA analysis; phase transformations and transitions could be observed up to 100 °C (permissible operating temperature of biodegradable plastics) through deviations from the normal expansion curve (linear). Thermogravimetric analysis confirmed the possibility of use in very good conditions, without mass loss up to temperatures of 200 °C and even a little higher than that.

Thus, the obtained results highlighted a series of functional characteristics of biodegradable lignin-based materials—Arboform^®^ LV3 Nature, Arboblend^®^ V2 Nature, Arbofill^®^ Fichte and Arboform^®^ LV3 Nature reinforced with aramid fibers, which offers the possibility of the progressive substitution of a series of fossil-based plastics from various industrial branches: according to the mechanical analysis in dynamic regime: PP, PP reinforced with glass fiber, short glass fiber, nanoclay, LDPE, LLDPE, HDPE, etc.

- From the thermal expansion and thermogravimetric point of view, the studied biodegradable materials behave like classical polymers, with the only difference being the possibility of working at high temperatures, as most become thermally unstable at temperatures above 200 °C. However, they exhibit their applicability, depending on the necessary functional requirements, even in these conditions, in a representative series of applications.

The purpose of the structural and morphological analysis by SEM and EDX was to confirm the adhesion and the uniformity of the connections between the fibers and the matrix. Aramid fibers are evenly distributed throughout the biopolymer structure, with their size being of the micrometers order. The large amount of oxygen and carbon in their chemical structure strengthened the claim that they are highly biodegradable. FT-IR analysis confirms the incorporation of amide fibers, due to the highlighted amide groups.

## Figures and Tables

**Table 1:** Main characteristics/properties of the studied materials [8,9,10].

	Material	Test Method	Arboform^®^ LV3 Nature	Arboblend^®^ V2 Nature	Arbofill^®^ Fichte
Characteristic					
MVR (190 °C/2.16 kg) (cm^3^/10 min)		DIN EN ISO 1133	13	15	6
HDT/B, A (°C)		ISO 75	49.8	50.4	111
Density (g/cm^3^)		DIN EN ISO 1183	1.29	1.25	1.03
Tensile strength (MPa)		DIN EN ISO 527	37	22.8	33
Tensile strain at break (%)		DIN EN ISO 527	0.6	11.8	4.1
Impact strength (+23 °C) (kJ/m^2^)		ISO 179/1eU	11	58	17

Note: MVR—melt volume rate; HDT—heat deflection temperature.

**Table 2:** Main technological parameters used at injection molding.

Process Parameters	Injection Temperature (°C)	Injection Pressure (MPa)	Injection Time (s)	Cooling Time (s)	Injection Speed (m/min)
Arboform^®^ LV3 Nature	160	100	11	25	80
Arboblend^®^ V2 Nature	170	110	6	25	90
Arbofill^®^ Fichte	155	90	9	25	80
Arboform^®^ LV3 Nature reinforced with aramid fibers	175	100	11	25	80

**Table 3:** Thermogravimetric characteristics of the studied materials.

Material	Thermal Degradation Stages	T_onset_ (°C)	T_peak_ (°C)	T_endset_ (°C)	W (%)	Residue (%)
Arbofill^®^ Fichte	I	89	114	139	1.33	5.87
	II	267	368	377	20.23	
	III	417	463	486	71.76	
Arboblend^®^ V2 Nature	I	278	332	354	74.61	3.64
	II	379	401	419	21.08	
Arboform^®^ LV3 Nature	I	40	39	78	2.15	11.31
	II	262	335	369	82.77	
	III	552	577	595	2.54	
Arboform^®^ LV3 Nature reinforced with aramid fibers	I	37	62	102	1.37	10.55
	II	139	164	192	2	
	III	280	334	369	83.62	
	IV	546	576	584	2.24	

Note: T_onset_—the temperature at which thermal degradation begins; T_endset_—the temperature at which the thermal degradation ends; T_peak_—the temperature at which the rate of degradation is maximum, extracted from the DTG curve peak; W%—mass percentage loss; residue—the amount of degraded sample remaining at a temperature above 700 °C.

## Data Availability

The data presented in this study are available on request from the corresponding author.
